# Bayesian hierarchical modeling of means and covariances of gene expression data within families

**DOI:** 10.1186/1753-6561-1-s1-s111

**Published:** 2007-12-18

**Authors:** Roger Pique-Regi, John Morrison, Duncan C Thomas

**Affiliations:** 1 Department of Preventive Medicine, University of Southern California, 1540 Alcazar Street, CHP-220, Los Angeles, California 90089, USA

## Abstract

We describe a hierarchical Bayes model for the influence of constitutional genotypes from a linkage scan on the expression of a large number of genes. The model comprises linear regression models for the means in relation to genotypes and for the covariances between pairs of related individuals in relation to their identity-by-descent estimates. The matrices of regression coefficients for all possible pairs of single-nucleotide polymorphisms (SNPs) by all possible expressed genes are in turn modeled as a mixture of null values and a normal distribution of non-null values, with probabilities and means given by a third-level model of SNP and trait random effects and a spatial regression on the distance between the SNP and the expressed gene. The latter provides a way of testing for *cis *and *trans *effects. The method was applied to data on 116 SNPs and 189 genes on chromosome 11, for which Morley et al. (*Nature *2004, 430: 743–747) had previously reported linkage. We were able to confirm the association of the expression of *HSD17B12 *with a SNP in the same region reported by Morley et al., and also detected a SNP that appeared to affect the expression of many genes on this chromosome. The approach appears to be a promising way to address the huge multiple comparisons problem for relating genome-wide genotype × expression data.

## Background

Recent advances in genomic technology now allow genotyping of hundreds of thousands of single-nucleotide polymorphisms (SNPs) and measurement of the expression of tens of thousands of genes on single microarrays or chips at a manageable cost. Extensive literature on the analysis of gene expression data has evolved over the last five years, and since the advent of ultra-high-volume genotyping platforms, genome-wide association and linkage scans using SNPs have also become feasible. The multiple comparisons problem is central to the analysis of either type of high-volume data. In 2001, Jansen and Nap [1] proposed combining the analysis of the two technologies in what he called "genetical genomics" to provide insight into the genetic regulation of gene expression.

However, only quite recently have attempts been made to relate the two technologies, first by Morley et al. [2] in a *linkage *scan for 3554 expressed genes in relation to 2756 autosomal SNP markers, and subsequently by the same group [3] in a genome-wide *association *scan of 27 of the expressed genes with the highest linkage in the first study, in relation to >770,000 SNPs. (See also Schadt et al. [4] and Stranger et al. [5] for similar analyses.) Independently, Tsalenko et al. [6] proposed a biclustering method to visualize SNPs and the transcripts they regulate, using an approach that is more visual than statistical. The multiple comparisons problem in such analyses (2.7 billion comparisons in the association analysis) dwarfs those from either genome-wide linkage or association analyses of single traits or supervised cluster analyses of expression data in relation to single outcomes.

Therefore, there is a need to develop new statistical methods to analyze all transcripts and genotypes together. Here, we describe a novel hierarchical Bayesian approach to the analysis of all possible pairs of associations and linkages between expressed genes and SNP markers. We demonstrate the results for chromosome 11 and we argue that the method can be extended to cover the entire genome and transcriptome.

## Methods

### Statistical model

Let *Y*_*ij*_^*n *^denote the expression of gene *n *in member *j *of family *i *and let *G*_*ij*_^*m *^be the corresponding SNP genotype at marker *m *at location *x*_*m*_. For the means and covariances of the expression traits, we adopted a generalized estimating equations model of the form used by Thomas et al. [7]

*E*(*Y*_*ij*_^*n*^) ≡ μ_*ij*_^*n *^= α_0_^*n *^+ Σ_*m *_*A*^*nm *^*G*_*ij*_^*m *^

*E*(*C*_*ijk*_^*n*^) ≡ χ_*ijk*_^*n *^= β_0_^*n *^+ *B*^*n*^*Z*_*ijk*_(*X*^*n*^),

where *C*_*ijk*_^*n *^= (*Y*_*ij*_^*n *^- μ_*ij*_^*n*^)(*Y*_*ik*_^*n *^- μ_*ik*_^*n*^) and *Z*_*ijk*_(*x*) are the estimated *E*(*IBD*_*ijk*_(*x*)|**G**_*i*_) at chromosomal location *x *for pairs (*j*, *k*) from nuclear family *i*, based on the complete multilocus marker data. *X*^*n *^is a latent variable for location of the unobserved causal locus linked to expression trait *n*. For *j *= *k*, *V*(*Y*_*ij*_^*n*^) = χ^*n *^models the gene expression variance in Eq. (2).

In Eq. (1), the regression coefficients *A*^*nm *^are modeled as a mixtures of null values with probabilities 1-π^*nm *^and a normal distribution of non-null values with means α^*nm *^expressed in terms of row and column effects:

*A*^*nm *^~ (1 - π^*nm*^) δ (**0**) + π^*nm *^*N*(α^*nm*^, σ^2^),

where

α^*nm *^= γ_0_^*A *^+ γ_1_^*A *^I(*x*_*m *_∈ *R*_*n*_) + *e*_*m*_^*A *^+ *h*_*n*_^*A*^

logit(π^*nm*^) = γ_0_^*P *^+ γ_1_^*P *^I(*x*_*m *_∈ *R*_*n*_) + *e*_*m*_^*P *^+ *h*_*n*_^*P*^.

The parameter γ_1 _distinguishes between *cis *and *trans *effects, a *cis *interaction occurs when the chromosomal location *x*_*m *_of SNP *m *is within the interval *R*_*n*_, the alignment region for the gene expression probe *n*. The random effects **e **and **h **are distributed as

(*e*_*m*_^*A*^, *e*_*m*_^*P*^) ~ *N*_2_(**0**, **T**)

(*h*_*n*_^*A*^, *h*_*n*_^*P*^) ~ *N*_2_(**0**, **W**)

and the γs, **T**, **W **have uninformative normal and Wishart priors.

The regression coefficients *B*^*n *^in the covariance model in Eq. (2) are handled similarly, except that we assume each trait has at most one region linked to it. (This is not essential to the method, because Eq. (2) could be extended to a summation over multiple independent linkage regions, but it would not make sense to offer all marker locations simultaneously, since the IBD variables are highly correlated from one location to the next.) Thus, we assume

**Figure 1:**
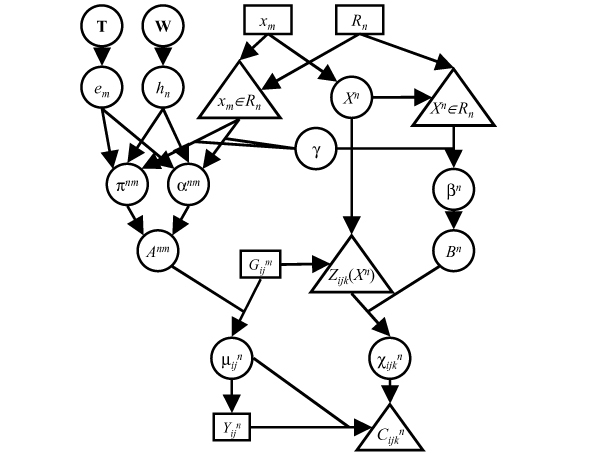
**Directed acyclic graph for the analysis model**. Squares represent observed data, circles represent parameters or latent variables, triangles represent deterministic nodes.

*B*^*n *^~ *N *[γ_0_^*B *^+ γ_1_^*B *^I(*X*^*n *^∈ *R*_*n*_), τ^2^]

and pick a uniform prior on *X*^*n*^; to simplify the calculations, we restrict *X*^*n *^to the observed marker locations *x*_*m *_and compute IBD probabilities only at these locations. Thus, *X*^*n *^has a discrete distribution with prior masses inversely proportional to the local marker density, here estimated simply as |*x*_*m*+1 _- *x*_*m*-1_|. The full model is represented in the directed acyclic graph shown in Figure 1.

We fitted the model using a Markov-chain Monte Carlo (MCMC) approach, implemented in Matlab. Updates of all parameters except the location parameters *X*^*n *^values involve standard Gibbs sampling from their respective full conditional distributions, e.g., [α_0_^*n*^|**Y**^*n*^, **G**, **A**], [β_0_^*n*^|**C**^*n*^, **Z**, **B**], [**A**^*nm*^|**Y**^*n*^, **G**^*m*^, α_0_^*n*^, π^*nm*^, α^*nm*^, σ^2^], etc. The updates of the *X *values are based on a Metropolis-Hastings procedure with a random walk proposal. The sequence was started ten times from several initial points chosen from an overdispersed prior around rough estimates. Half of the initial samples are discarded and the second half is kept. The number of kept samples, *L *= 4000, is chosen to be large enough so that for all parameters of interest the variance between sequences *V*_*B *_is comparable to that within sequence *V*_*W*_, *R *< 1.10:

$$\hat{R}=\sqrt{\frac{L-1}{L}+\frac{1}{L}\frac{V_{B}}{V_{W}}}.$$

The rationale behind this convergence monitoring procedure is described and justified by Gelman et al. [8].

### Subjects, genotypes, and phenotypes

In order to keep the computation to a manageable level, we restricted this analysis to the SNP genotypes and expressed genes on chromosome 11, since previous analyses by Morley et al. [2] had found evidence of both *cis *and *trans *linkages at this chromosome. The final data set thus had 116 SNPs and 189 expressed genes. IBD status was estimated from the complete two-generation pedigrees (excluding grandparents) by a program written by JM based on the Lander-Green algorithm [9]. All 378 sib pairs (110 individuals) from the available 14 families were included in the phenotype analysis.

## Results

**Figure 2:**
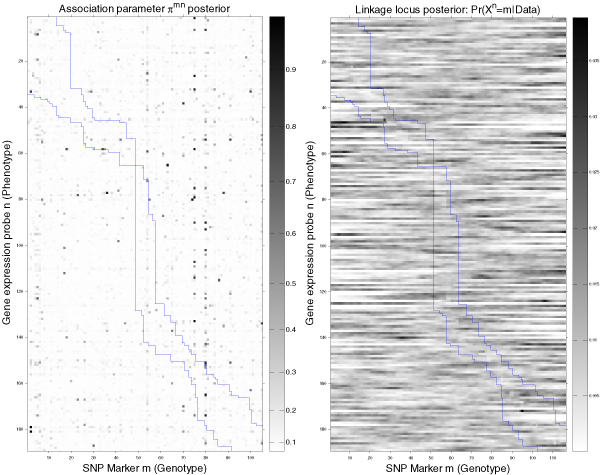
**Gene expression × Genotype associations and residual linkage summary**. Left, Image describing the mean value of the association parameters π^nm ^between the gene expression phenotypes (rows) and the SNP genotypes (columns). The matrix shows that the interactions are very sparse (dark spots), meaning that phenotypes are controlled by small number of SNPs, with no apparent concentration along the *cis *region delimited by blue lines. However, there exist some SNPs (columns) that seem to be correlated with a large set of phenotypes, potentially indicating a master regulatory region. Right, Image describing the posterior probability of the linkage locus after removing the association effect from the covariance.

After convergence has been reached, the number of regression coefficients with nonzero coefficients in Eq. (1) is very small. This is because in the mixture model employed in Eq. (3), a large number of the probabilities are close to 0 (Figure 2).

**Figure 3:**
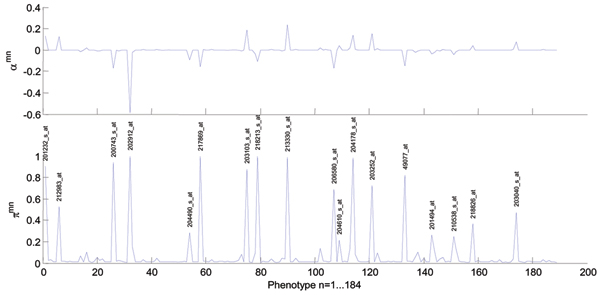
**Potential master regulatory region around rs916482 SNP**. Bottom plot is the cross-section of column 84 of Figure 1, describing the association between all phenotypes in chromosome 11 and SNP m = rs916482. The top plot shows the sign of dependence on the genotype. This SNP has a large number of associated genotypes, providing a strong indication of a master regulatory region.

**Figure 4:**
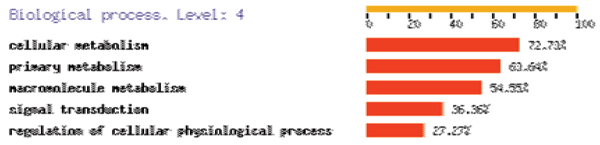
**Gene ontology on potential master regulatory region**. Overrepresented GO terms by the phenotypes associated to the SNP rs916482 analyzed using FatiGO .

Figure 2 also shows that each gene expression phenotype is explained by relatively few genotypes that have a role in regulating their expression. Table 1 lists, for the best predicted phenotypes, the SNPs included most frequently in the model. Significantly, the top ranking phenotype, *HSD17B12 *(217869_at), associated with SNP rs1453389, is the same as the one reported by Cheung et al. as associated with another SNP in the same region (not included in the GAW data set). Figure 3 shows that some SNPs in chromosome 11, especially rs916482, are significantly associated with more phenotypes than others. These SNP may be within a master regulatory region of gene expression. The list of gene ontology terms that were over-represented in the list of its associated genes involved mostly metabolic functions (Figure 4).

**Figure 5:**
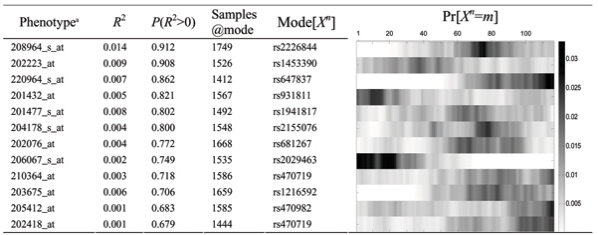
**Linkage of residual gene expression variation after association.**  Phenotypes ranked by most significant coefficient of determination in the covariance model, the posterior distribution of their locus position *X_n_*, and its mode. The coefficient of determination and its significance are calculated from samples drawn around the mode (10%).

**Table 1:** Top-ranking associations

				Top SNPs used in the prediction							
				
Phenotype^a^	Probe	*R*^2^	*P*(*R*^2 ^> 0)	SNP1	π^*nm*^	SNP2	π^*nm*^	SNP3	π^*nm*^	SNP4	π^*nm*^
*HSD17B12*	217869_at	0.25	0.988	rs1453389^b^	1.00	rs916482	1.00	rs1425151	0.40	rs509628	0.28
*C11orf10*	218213_s_at	0.12	0.986	rs916482	1.00						
*AMPD3*	207992_s_at	0.19	0.985	rs2029463^b^	0.81	rs948215	0.80	rs1157659	0.21	rs1491846	0.17
*FEZ1*	203562_at	0.12	0.984	rs2029463	1.00	rs2155076	0.20	rs948215^b^	0.11		
*ADM*	202912_at	0.11	0.982	rs916482	1.00						
*STIP1*	213330_s_at	0.11	0.981	rs916482	0.99	rs1319730	0.33				
*DDB1*	208619_at	0.15	0.978	rs1530966	0.91	rs597345	0.54	rs1499511	0.10		
*FADS1*	208964_s_at	0.14	0.974	rs1216592	0.85	rs1605026	0.38	rs591804	0.35		
*TPP1*	200743_s_at	0.13	0.970	rs916482	0.94	rs1157659	0.14	rs902215^b^	0.14		
*RBM14*	204178_s_at	0.10	0.966	rs916482	0.98	rs674237	0.10				
*HMBS*	203040_s_at	0.13	0.963	rs86392	0.49	rs916482	0.47	rs1319730^b^	0.44	rs1945906	0.20
*PPME1*	49077_at	0.11	0.958	rs916482	0.82	rs2155001	0.16				
*CD44*	204490_s_at	0.12	0.957	rs702738	0.34	rs916482	0.28	rs1319730	0.28	rs1453390^b^	0.17
*NRGN*	204081_at	0.10	0.946	rs2029463	0.93	rs961746	0.16	rs509628	0.15		
*NDUFS8*	203190_at	0.11	0.944	rs86392	0.68	rs1319730	0.33	rs1945906	0.32		
*PSMD13*	201232_s_at	0.09	0.923	rs916482	0.91	rs1319730	0.12				

^a^Phenotypes ranked by most significant coefficient of determination, and some of their top associated SNPs ranked by average π^*nm*^.

^b^*cis*-acting interaction, defined as the SNP being within 10 MB of the phenotype probe alignment.

The covariance model [Eq. (2)] results are summarized in the right panel of Figure 2, and the strongest linkage peaks are listed in Table 2. This linkage is for the remaining variation not explained by the association/means model [Eq. (1)], and the peaks would correspond to unseen genotypes that are in LD with a marker that was not used in the association model. Thus, this explains in part why linkage results are less compelling than the association ones. However, for those phenotypes for which significant linkage was found, the expression covariance increased with the IBD status, especially in 208964_s_at.

**Table 2:** Linkage of residual gene expression variation after association

Phenotype^a^	*R*^2^	*P*(*R*^2 ^> 0)	Samples @mode	Mode [*X*^*n*^]	Pr [*X*^*n *^= *m*]
208964_s_at	0.014	0.912	1749	rs2226844	
202223_at	0.009	0.908	1526	rs1453390	
220964_s_at	0.007	0.862	1412	rs647837	
201432_at	0.005	0.821	1567	rs931811	
201477_s_at	0.008	0.802	1492	rs1941817	
204178_s_at	0.004	0.800	1548	rs2155076	
202076_at	0.004	0.772	1668	rs681267	
206067_s_at	0.002	0.749	1535	rs2029463	
210364_at	0.003	0.718	1586	rs470719	
203675_at	0.006	0.706	1659	rs1216592	
205412_at	0.001	0.683	1585	rs470982	
202418_at	0.001	0.679	1444	rs470719	

^a^Phenotypes ranked by most significant coefficient of determination in the covariance model, the posterior distribution of their locus position *X*^*n*^, and its mode. The coefficient of determination and its significance are calculated from samples drawn around the mode (10%).

## Discussion

We have introduced a novel hierarchical Bayes model for genetic control of gene expression. Our approach to dealing with the multiple comparisons problem is to represent the matrices of all possible SNP × expressed gene association or linkage coefficients in terms of row and column random effects, along with a spatial regression on the distance between the two. Although this allows inference on specific pairs, we have greater interest in the variances of the row and column effects, which reflect systematic tendencies for SNPs to affect variable numbers of phenotypes and for phenotypes to be differentially expressed. Our mixture model also supports the possibility that the vast majority of such associations or linkages would be truly null, and allows separate estimation of both the probability and magnitude of non-null tests. So far we have not imposed any relationship between the parameters of the association (means) and linkage (covariance) models, but one might contemplate using the broad regions where linkage is seen for a particular phenotype as a prior for testing single-SNP associations with that phenotype.

The strongest gene-expression × SNP association reported by Cheung et al. [3] on chromosome 11 also appeared in our results as the most significant association, but with a SNP close to theirs (their reported SNP was not included in the data set). We also found evidence of at least one SNP that appears to be linked to a large number of expressed genes, suggesting the existence of master regulatory genes in that region.

We chose to restrict these analyses to a subset of genes and SNPs on a single chromosome to test the feasibility of the method. In principle the approach could be applied on a genome-wide scale, since the computation time increases linearly with *m*, *n*, sample size, and number of MCMC samples. Generating 4000 MCMC samples required 6 hours on a 2.2 GHz single-processor machine. However, one outstanding methodological challenge that would have to be addressed before the approach could be applied to dense SNP associations would be how to deal with the multicollinearity problem; for this reason, we chose to restrict this analysis to only a subset of SNPs that were not in strong LD with each other.

## Competing interests

The author(s) declare that they have no competing interests.
